# Association of leukocyte count with death in people with HIV: A longitudinal study over 24 years

**DOI:** 10.1371/journal.pone.0340678

**Published:** 2026-01-08

**Authors:** Zoe Klein, Sophia C. Meyer, Isabella C. Schoepf, Marc Weber, Christian W. Thorball, Lene Ryom, Neeltje A. Kootstra, Peter Reiss, Dominique L. Braun, Maria C. Thurnheer, Catia Marzolini, Christian R. Kahlert, Enos Bernasconi, Matthias Cavassini, Annalisa Marinosci, Jacques Fellay, Roger D. Kouyos, Huldrych F. Günthard, Bruno Ledergerber, Philip E. Tarr, and the Swiss HIV Cohort Study

**Affiliations:** 1 University Center for Internal Medicine and Infectious Diseases Service, Kantonsspital Baselland, Department of Clinical Research, University Hospital Basel, University of Basel, Bruderholz, Switzerland; 2 Hepatology, Department for Visceral Surgery and Medicine, Bern University Hospital, University of Bern, Bern, Switzerland; 3 Department of Infectious Diseases and Hospital Epidemiology, University Hospital Zurich, University of Zurich, Zurich, Switzerland; 4 Precision Medicine Unit, Lausanne University Hospital and University of Lausanne, Lausanne, Switzerland; 5 Centre of Excellence for Health, Immunity, and Infections, Rigshospitalet, University of Copenhagen, Copenhagen, Denmark; 6 Department of Infectious Diseases, Hvidovre University Hospital, Copenhagen, Denmark; 7 Department of Clinical Medicine, University of Copenhagen, Denmark; 8 Experimental Immunology, Amsterdam University Medical Centers, University of Amsterdam, Amsterdam, The Netherlands; 9 Amsterdam University Medical Centers, University of Amsterdam, Global Health, and Amsterdam Institute for Global Health and Development, Amsterdam, The Netherlands; 10 Institute of Medical Virology, University of Zurich, Zurich, Zurich, Switzerland; 11 Department of Infectious Diseases, Bern University Hospital, University of Bern, Bern, Switzerland; 12 Division of Infectious Diseases and Hospital Epidemiology, University Hospital Basel, Basel, Switzerland; 13 Division of Infectious Diseases, Kantonsspital St Gallen, St. Gallen, Switzerland; 14 Division of Infectious Diseases, Ospedale Regionale Lugano, University of Geneva and Università della Svizzera italiana, Lugano, Switzerland; 15 Infectious Diseases Service, Lausanne University Hospital, University of Lausanne, Lausanne, Switzerland; 16 Division of Infectious Disease, Geneva University Hospital, Geneva, Switzerland; 17 School of Life Sciences, Ecole Polytechnique Fédérale de Lausanne, Lausanne, Switzerland; Children's National Hospital, George Washington University, UNITED STATES OF AMERICA

## Abstract

**Objectives:**

Increased blood leukocytes are associated with all-cause mortality and death from coronary disease and cancer in the general population. Limited information is available in people with HIV.

**Methods:**

We analyzed blood leukocytes in 1850 participants of the Swiss HIV Cohort Study who died (2000–2023) and 1850 matched controls (median age at death 52 years, 77% male, 77% with suppressed HIV RNA on antiretroviral therapy). We assessed uni-/multivariable odds ratios (OR) for all-cause mortality, considering major clinical and HIV-related mortality risk factors, leukocytes measured >1 year before death (primary analysis) and in the latest available blood sample, and potential confounders for leukocytes.

**Results:**

Leukocytes showed a U-shaped association with mortality. At a median of 433 (interquartile range [IQR], 396–495) days before death, multivariable-adjusted OR for death in the highest (leukocytes ≥7730/μL) vs. middle quintile (leukocytes 5290–6260/μL) was 1.56 (95% confidence interval, 1.20–2.02). Multivariable-adjusted OR in the lowest (leukocytes ≤4250/μL) vs. middle leukocyte quintile was 1.51 (1.14–2.01). For comparison, death-OR for hypertension, diabetes and current smoking were 1.27 (1.06–1.53), 1.91 (1.41–2.57), and 2.72 (2.14–3.45), respectively. Leukocytosis was uncommon (cases, 4.4% vs. controls, 2.3%; p = 0.004). The effect size of the highest leukocyte quintile increased in the latest blood sample (median 86 [IQR], 43–152 days before death; OR=1.99 [1.44–2.76]). High leukocytes were associated with death from non-AIDS/non-hepatic cancer, cardiovascular, and respiratory causes. Low leukocytes were associated with liver-related death.

**Conclusions:**

High leukocytes, measured >1 year before death and mostly within the normal range, are independently associated with mortality in people with HIV in Switzerland.

## Introduction

Ever since the introduction of highly active antiretroviral therapy (ART), mortality of people with HIV has dramatically decreased. Still, differences in life expectancy of people with HIV compared to the general population persist [1–4]. Reasons for increased mortality in HIV despite suppressive ART include higher rates of hepatitis C co-infection, increased rates of drug use and smoking [5–7]. In addition, extensive inflammatory biomarker literature in HIV documents chronic systemic, residual inflammation as a key mechanism in the development of major non-AIDS conditions including diabetes, coronary artery disease, and certain cancers [8–11].

A well-known, routinely measured biomarker of inflammation is blood leukocytes. The role of different leukocyte subsets in the pathogenesis of HIV infection is well established, including most notably the role of progressive CD4 decline with opportunistic complications of HIV and with mortality [12–14]. In HIV infection, neutrophils are capable of both activating and suppressing T-lymphocytes [15,16]. Macrophages may be infected by HIV, contribute to viral persistence, and via their migration, contribute to spread of HIV to different body tissues. In addition, macrophages and biomarkers of macrophage activation (e.g., soluble CD14, soluble CD163) may contribute to chronic systemic inflammation and HIV-associated, aging-related comorbidities [17–21]. CD8 cells are believed to be crucial in controlling HIV infection via various mechanisms, e.g., HIV-specific cytotoxic activity or suppression of HIV replication through soluble factors [22].

We recently showed an independent association of high total blood leukocyte levels, mostly within the normal range, and both coronary events and new onset diabetes mellitus in people with HIV in Switzerland [11,23]. Leukocytes were elevated years before coronary events and diabetes in people with HIV [11,23], consistent with meta-analysis in the general population [24]. This shows that the direction of the effect is from inflammation to coronary events and diabetes and argues against potential reverse causality.

In the general population, an association between elevated leukocytes and increased all-cause, coronary, and cancer mortality has been well recorded since the 1980s [25–27]. In people with HIV, evidence to support an association of leukocytes with mortality is limited. Researchers with the Veterans Aging Cohort Study (VACS) and the ART-Cohort Collaboration showed that adding leukocyte count to the original VACS Index improved prediction of mortality over 5 years follow-up [28].

The aim of this study therefore was to further investigate any independent association between total leukocyte levels and all-cause mortality in participants of the Swiss HIV Cohort Study (SHCS) over a 24-year observation period, in the context of relevant traditional and HIV-related risk factors. Because our main interest was in analyzing whether blood leukocytes can predict death, the primary analysis included leukocytes measured >1 year before death.

## Methods

### Study population, study periods

We included people with HIV enrolled in the SHCS (www.shcs.ch, [29]) who provided written informed consent and had ≥ 1 year of prospective SHCS follow-up. The research project was approved by the Cantonal Ethics Commission Zurich, Section B (Prof. em. Dr. med. Konrad E. Bloch) on the 26^th^ April 2024 (Application BASEC-Nr.: 2023–02080). All participants provided written informed consent. Regarding the main analysis (all-cause mortality), cases died and controls were alive during the study period (1.1.2000–31.12.2023).

### Case-control matching

We selected 1 control per case using incidence density sampling [30], to account for differences in antiretroviral therapy use and other differences during different observation periods. Matching date was the date of death of the corresponding case. Controls were matched on similar observation duration and during similar calendar periods. Matching criteria were date of SHCS registration ±2 years and age at matching date ±2 years. Because CD4 cells are in part collinear with leukocytes we did not match on CD4 count. Because the majority of SHCS participants are male and because in preliminary analysis matching on sex at birth resulted in loss of significant numbers of female cases due to no controls available, we did not match on sex at birth. Duration of observation was until death for cases and until matching date for controls, respectively.

### Power calculation

With 1600 cases and 1 control per case, we have a power of 0.9 to detect odds ratios of ≥1.3, assuming an exposure correlation in the case-control set of 0.2 between pairs [31].

### Leukocyte count

While infectious illness might be associated with increased leukocytes and with death in the short term, we hypothesized that high leucocytes are associated with mortality when routinely measured already one or more years before death. Similar to our previous studies on high leukocytes and coronary events and diabetes [11,23], we therefore considered in the primary analysis only leukocytes measured per protocol (at 6-monthly SHCS visits) and 1–5 years before death. Within this time window we selected the most recent (i.e., closest to matching date) leukocyte measurement.

### Clinical risk factors for death

We included covariables known to be associated with death in the general population and/or in people with HIV, as we did previously [1,23,32]. We ascertained covariables at the time of leukocyte measurement (except CD4 nadir: lowest CD4 value during study period). Covariables included HIV acquisition mode, ethnicity, highest educational level attained, body mass index (BMI [kg/m^2^]; underweight, normal, overweight, obese), smoking (never/current/past), hypertension (blood pressure ≥140/90 mmHg or use of antihypertensive medication), diabetes (plasma glucose ≥7.0 mmol/L [fasting] or ≥11.1 mmol/L [nonfasting] and typical symptoms or plasma glucose ≥11.1 mmol/L, 2 hours after oral intake of 75g glucose or HbA1c ≥ 6.5% [33]), and family history of coronary artery disease (CAD). HIV-related co-variables included viral load (HIV RNA < / ≥ 50 copies/mL), hepatitis C [34] and cytomegalovirus (CMV) seropositivity [35] closest to the time of leukocyte measurement. Alcohol intake was classified as none/mild vs moderate/heavy; defined in the SHCS until 2012 as </ ≥ 40 g [men], < / ≥ 20 g [women]), and using the Alcohol Use Disorders Identification Test-C questionnaire beginning in 2013 (</ ≥ 4 points [men], < / ≥ 2 points [women]) [36]. Dyslipidemia was defined as total cholesterol >6.2 mmol/L or HDL < 1 mmol/L [men] and <1.2 mmol/L [women] or use of lipid-lowering drugs [37].

### Potential confounding variables associated with Leukocyte count

We tested each variable plus CD4 count category closest to the time of leukocyte measurement (<200, 200–349, 350–499, 500+) in the model in univariable and bivariable analyses for potential confounding (e.g., OR for death for leukocyte quintiles with/without the potential confounder; **S2 Table**). We did not analyze non-HIV inflammatory conditions or corticosteroid use due to insufficient information (e.g., specific diagnoses, corticosteroid duration/dose/dates).

### Sensitivity analyses

To assess the robustness of the leukocyte-death association we performed several sensitivity analyses, including; 1) analysis without smoking and 2) analysis without obesity in the model, because smoking [11,25,38–40] and obesity are associated not only with mortality but also with increased leukocytes [41,42]; 3) analysis excluding participants with leukocytosis; 4) analysis including only participants with suppressed HIV-RNA; 5) analysis including dyslipidemia and alcohol intake; 6) analysis at the latest available time point before matching date; 7) analysis including, in addition to leukocytes measured per protocol, also clinically indicated leukocyte measurements.

### Causes of death

Specific causes of death were analyzed for years 2005–2022, because the SHCS has collected detailed information about causes of death since 2005 using the Coding Causes of Death in HIV (CoDe) protocol [32,43]. We used the CoDe codes and 10 categories published by Weber et al in a recent SHCS analysis [43], reducing them to 7 categories by merging substance abuse/violent death, suicide/assisted suicide, other and unknown into a single category.

### Statistical analyses

Data were accessed for analyses on 24/07/2025. Characteristics of cases and controls were compared using Fisher’s exact test (categorical variables) and Wilcoxon’s rank-sum test (continuous variables). Univariable, bivariable and multivariable conditional logistic regression analyses were used to estimate associations of the different risk factors with death. We decided a priori to stratify leukocyte counts into quintiles for better visualization of potentially non-linear associations with death. Variables were entered into the multivariable model if their association in the univariable model had p < 0.2. We provide complete case analysis, i.e., we did not impute missing key data. Alcohol consumption was assessed in a sensitivity analysis. Because of significant collinearity between obesity and dyslipidemia (p < 0.01) we included dyslipidemia only in sensitivity analysis, but not in the primary analysis. Interactions were analyzed using likelihood ratio tests. The effect of potential confounders on the leukocyte-death association was tested on a 1:1 basis (bivariable models including interaction terms). We used Stata/SE 18.0 (StataCorp, College Station, TX, USA).

## Results

### Participants

**Fig 1:**
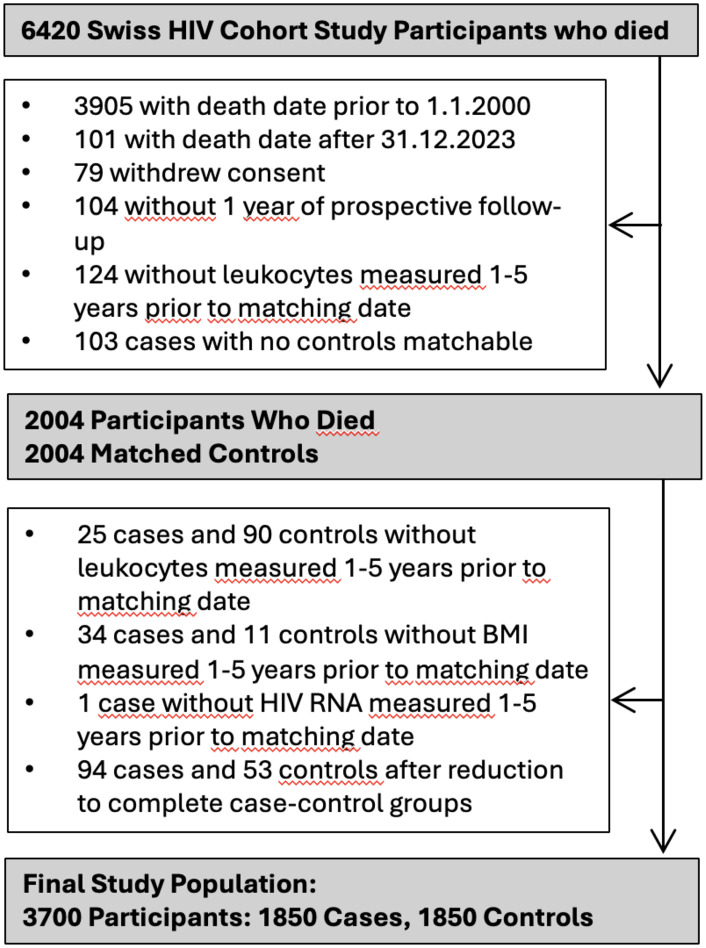
Study flowchart.

We included 3700 participants, i.e., 1850 cases who died during the study period and 1850 matched controls (**Fig 1**). **Table 1** shows characteristics of the participants. Cases were more likely to inject drugs, have no education beyond mandatory schooling, have underweight BMI, be current smokers, have hypertension, diabetes, chronic HCV infection, detectable HIV-viremia, CD4 < 350, CD4 < 200, CD4 nadir<50, and CDC stage C illness. Controls were more likely to be men who have sex with men (MSM), have overweight/obese BMI and undetectable HIV RNA (**Table 1**).

**Table 1:** Characteristics of Cases and Controls and Mortality Odds Ratio: Univariable and Multivariable Analysis.

		Participants				Analysis, Odds ratio (95% Confidence Interval); p-value	
		All Participants (n = 3700)	Cases (n = 1850)	Controls (n = 1850)	P-Value	Univariable^c^	Multivariable
Age (years), median (IQR)^d^		52 (44-61)	52 (44-61)	52 (44-61)	0.947^b^	^d^	^d^
Male sex, n (%)		2831 (76.5)	1415 (76.5)	1416 (76.5)	0.969^a^	Male sex: reference Female sex: 1.00 (0.86–1.17)	Male sex: reference Female sex: 0.63 (0.49–0.80)
Matching date, median (IQR)		28 March 2011 (21 October 2004 – 18 July 2017)	28 March 2011 (21 October 2004 – 18 July 2017)	28 March 2011 (21 October 2004– 18 July 2017)	1.000^b^	--	--
Duration of observation (years), median (IQR) ^d^		12.3 (7.1-18.6)	12.1 (6.8-18.4)	12.5 (7.4-19.0)	0.045^b^	^d^	^d^
HIV acquisition mode, n (%)	MSM	1478 (40.0)	569 (30.8)	909 (49.1)	<0.001^a^	(reference)	(reference)
	Injection drug use	1061 (28.7)	713 (38.5)	348 (18.8)		4.47 (3.64–5.49); p < 0.001	2.06 (1.46–2.89); p < 0.001
	Heterosexual	1033 (27.9)	506 (27.4)	527 (28.5)		1.61 (1.36–1.92); p < 0.001	1.65 (1.28–2.12); p < 0.001
	Other	128 (3.5)	62 (3.4)	66 (3.6)		1.57 (1.06–2.31); p = 0.024	1.34 (0.83–2.16); p = 0.236
Ethnicity, n (%)	White	3438 (92.9)	1733 (93.7)	1705 (92.2)	0.001^a^	(reference)	(reference)
	Black	148 (4.0)	81 (4.4)	67 (3.6)		1.20 (0.86–1.67); p = 0.294	1.46 (0.92–2.32); p = 0.108
	Hispanic	52 (1.4)	16 (0.9)	36 (2.0)		0.41 (0.22–0.77); p = 0.006	0.37 (0.17–0.83); p = 0.015
	Asian	62 (1.7)	20 (1.1)	42 (2.3)		0.45 (0.26–0.79); p = 0.005	0.60 (0.30–1.20); p = 0.149
Education, n (%)	Mandatory School	813 (22.0)	498 (26.9)	315 (17.0)	<0.001^a^	(reference)	(reference)
	Apprenticeship	1793 (48.5)	868 (46.9)	925 (50.0)		0.59 (0.50–0.70); p < 0.001	0.72 (0.57–0.90); p = 0.005
	Higher Education	830 (22.4)	342 (18.5)	488 (26.4)		0.43 (0.35–0.53); p < 0.001	0.72 (0.55–0.94); p = 0.016
	Other/Missing	264 (7.1)	142 (7.7)	122 (6.6)		0.78 (0.58–1.06); p = 0.111	0.75 (0.51–1.11); p = 0.148
BMI category, n (%)	Underweight	306 (8.3)	249 (13.5)	57 (3.1)	<0.001^a^	4.33 (3.18–5.89); p < 0.001	3.41 (2.35–4.95), p < 0.001
	Normal	2166 (58.5)	1081 (58.4)	1085 (58.7)		(reference)	(reference)
	Overweight	936 (25.3)	388 (21.0)	548 (29.6)		0.70 (0.59–0.82); p < 0.001	0.77 (0.63–0.94); p = 0.010
	Obese	292 (7.9)	132 (7.1)	160 (8.7)		0.81 (0.63–1.05); p = 0.107	0.76 (0.56–1.04); p = 0.084
Smoking status, n (%)	never	996 (26.9)	347 (18.8)	649 (35.1)	<0.001^a^	(reference)	(reference)
	current	1876 (50.7)	1138 (61.5)	738 (39.9)		3.48 (2.89–4.19); p < 0.001	2.72 (2.14–3.45); p < 0.001
	past	828 (22.4)	365 (19.7)	463 (25.0)		1.55 (1.27–1.88); p < 0.001	1.39 (1.09–1.77); p = 0.007
Hypertension, n (%)		1406 (38.0)	758 (41.0)	648 (35.0)	<0.001^a^	1.32 (1.15–1.52); p < 0.001	1.27 (1.06–1.53); p = 0.009
Diabetes		316 (8.5)	189 (10.2)	127 (6.9)	<0.001^a^	1.58 (1.24–2.01); p < 0.001	1.91 (1.41–2.57); p < 0.001
Family history of CAD		424 (11.5)	209 (11.3)	215 (11.6)	0.757^a^	0.97 (0.79–1.19); p = 0.756	--
Hepatitis C seropositivity, n (%)		1330 (36.0)	855 (46.2)	475 (25.7)	<0.001^a^	3.30 (2.76–3.91); p < 0.001	1.57 (1.19–2.09); p = 0.002
CMV seropositivity, n (%)		3093 (83.6)	1510 (81.6)	1583 (85.6)	0.001^a^	0.75 (0.63–0.89); p = 0.001	--
Leukocytes/μL, median (IQR)	One to five years before death (n = 3700)	5770 (4565-7300)	5700 (4250-7500)	5800 (4790-7030)	0.026^b^	Lowest vs. third leukocyte quintile: 2.45 (1.98–3.04); p < 0.001	Lowest vs. third leukocyte quintile: 1.51 (1.14–2.01); p = 0.004
						Highest vs. third leukocyte quintile: 1.82 (1.48–2.24); p < 0.001	Highest vs. third leukocyte quintile: 1.56 (1.20–2.02); p = 0.001
Leukocytosis (>11000/mL), n (%)		125 (3.4)	82 (4.4)	43 (2.3)	<0.001^a^	--	--
HIV RNA-status <50 copies/mL (undetectable), n (%)		2718 (73.5)	1245 (67.3)	1473 (79.6)	<0.001^a^	0.43 (0.36–0.52); p < 0.001	0.52 (0.41–0.65); p < 0.001
CD4 cell count one to five years before death (cells/μL), median (IQR)		464 (286-670)	377 (214-606)	530 (374-715)	<0.001^b^	--	--
CD4 cell count category one to five years before death (cells/μL), n (%)	0 – < 200	548 (14.8)	433 (23.4)	115 (6.2)	<0.001^a^	(reference)	(reference)
	200 – < 350	700 (18.9)	418 (22.6)	282 (15.2)		0.37 (0.28–0.49); p < 0.001	0.45 (0.32–0.62); p < 0.001
	350 – < 500	788 (21.3)	353 (19.1)	435 (23.5)		0.18 (0.14–0.24); p < 0.001	0.23 (0.17–0.32); p < 0.001
	≥500	1664 (45.0)	646 (34.9)	1018 (55.0)		0.13 (0.10–0.17); p < 0.001	0.17 (0.12–0.23); p < 0.001
CD4 nadir (cells/μL), median (IQR)		133 (49-237)	113 (40-212)	161 (63-263)	<0.001^b^	--	--
CD4 nadir <50 cells/μL, n (%)		926 (25.0)	544 (29.4)	382 (20.7)	<0.001^a^	1.61 (1.38–1.88); p < 0.001	--
Previous AIDS, n (%)		1291 (34.9)	797 (43.1)	494 (26.7)	<0.001^a^	--	--

**Note.** All data shown apply to the matching date and are number (%) of participants, unless otherwise indicated.

^a^Pearson’s Chi squared test.

^b^Wilcoxon rank-sum test.

^c^The same univariable death-ORs can be found in Supplementary S2 Table.

^d^Age, date of registration, and date of last follow-up visit in controls were matching criteria.

Abbreviations. CAD, coronary artery disease; CMV, cytomegalovirus; IQR, interquartile range; MSM, men who have sex with men.

### Leukocyte count, observed data

Median time between leukocyte measurement and death was 433 (IQR, 396–495) days in cases vs. 430 (IQR, 394–470) days in controls. Median leukocyte count >1 year prior to matching date was similar in cases vs. controls (5700 [IQR, 4250–7500] vs. 5800 [4780–7100], respectively; p = 0.018; **Table 1**). Leukocytosis (>11000/μL) was uncommon, but more frequently noted in cases vs. controls (4.4% vs. 2.3%; p = 0.004).

### Leukocyte count and death, univariable model

**Fig 2:**
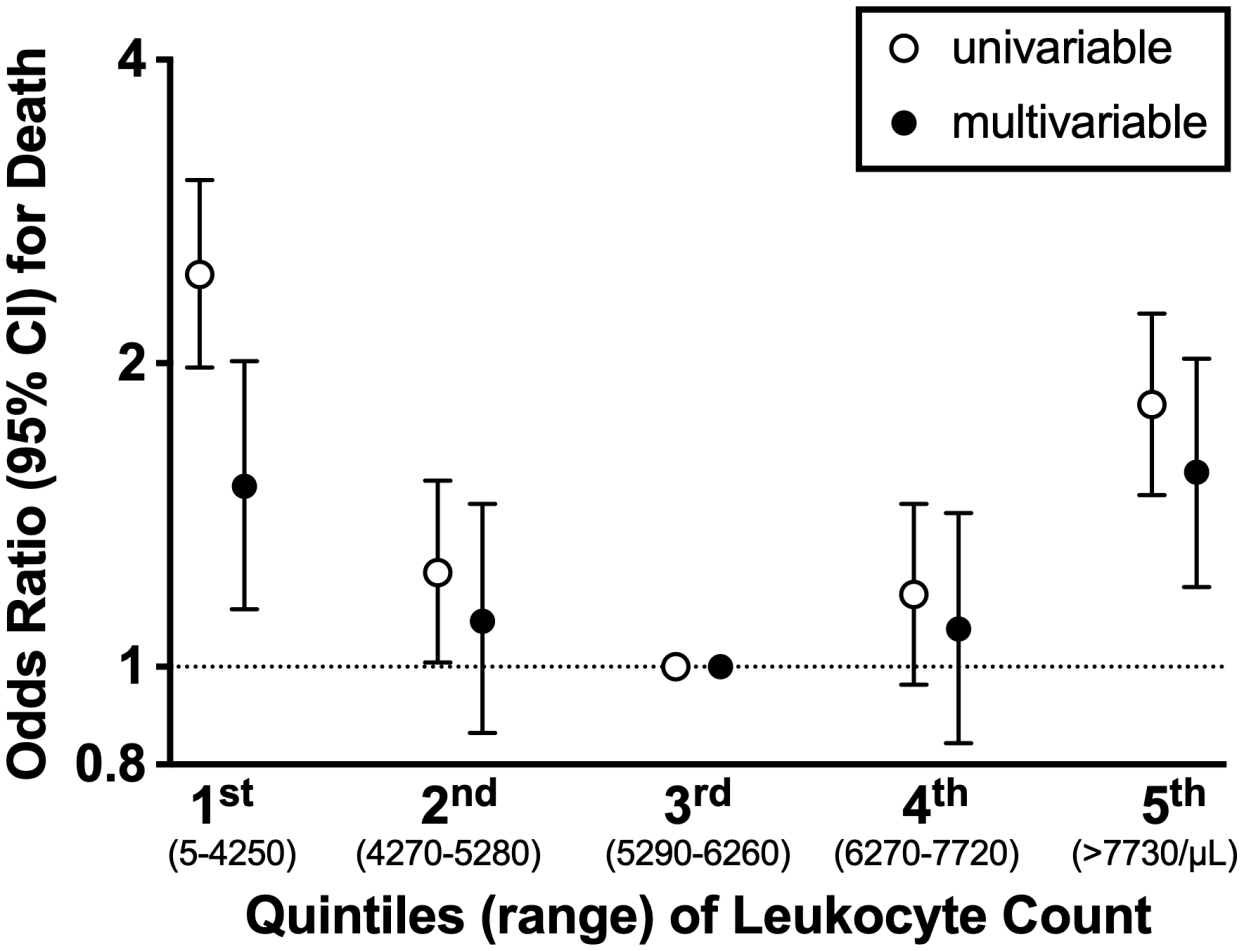
Association of Mortality with Blood Leukocyte Count, Measured 1 to 5 Years before Death, Odds Ratio and 95% Confidence Intervals [CIs]). Univariable and multivariable OR (with 95% CI) for first, second, third, fourth and fifth leukocyte quintile 1 to 5 years prior to death are shown. The third leukocyte quintile was used as reference. In univariable and multivariable analysis the lowest and highest leukocyte quintile were significantly associated with death. **Abbreviations**: CI, confidence interval.

**Fig 3:**
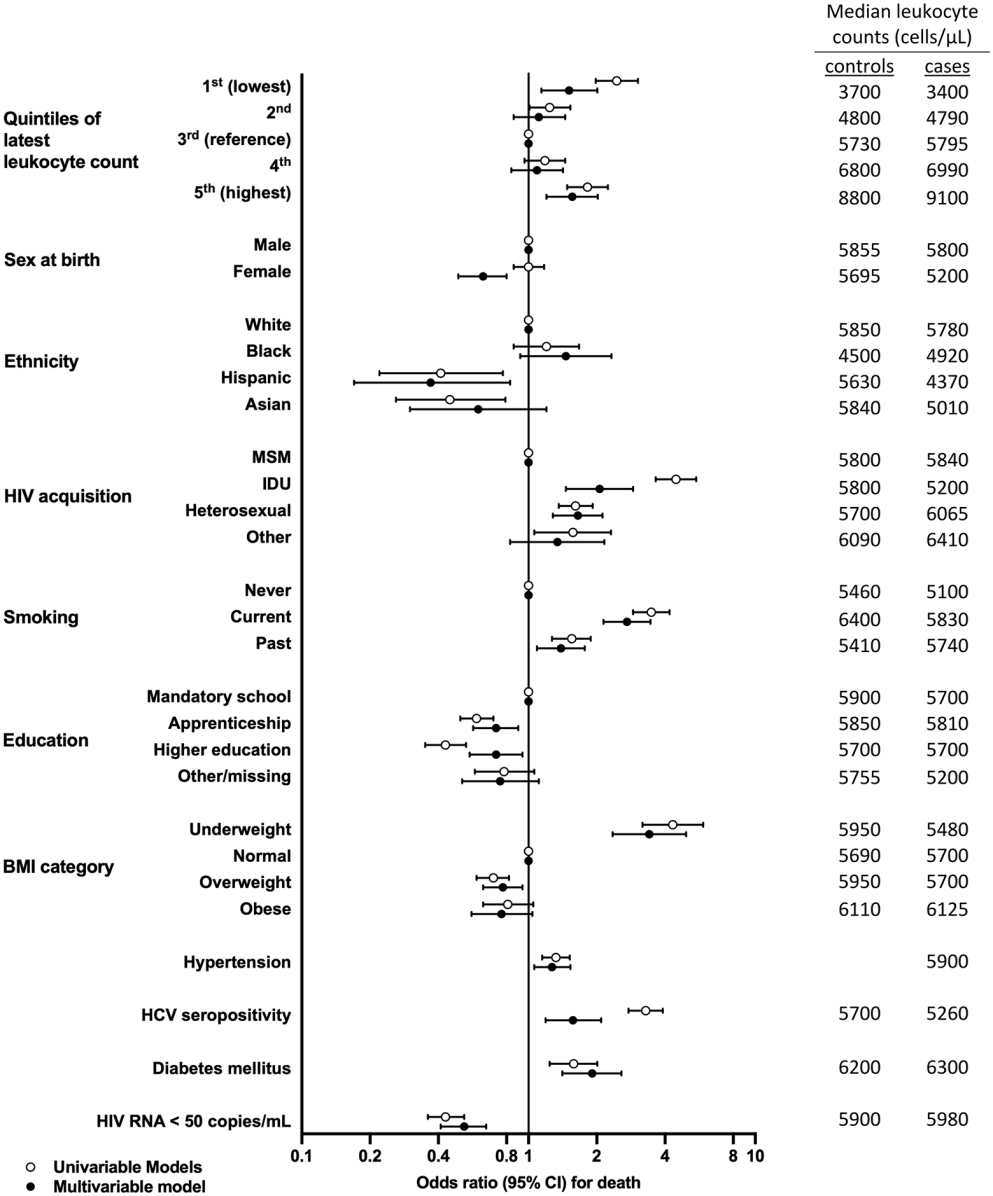
Odds Ratios (ORs) for Death (with 95% Confidence Intervals [CIs]), According to Clinical Risk Factors and Leukocyte Quintiles. Univariable and multivariable conditional logistic regression of associations of leukocyte count 1 to 5 years prior to matching date with death are shown for 1850 cases and 1850 controls. OR was significantly higher in the lowest vs. middle (reference) leukocyte quintile and highest vs. middle leukocyte quintile in univariable and multivariable analysis, adjusted for all variables shown. Note: **Table 1** shows all OR and 95% CIs illustrated in **Fig 3**. The table on the right-hand side of **Fig 3** shows median leukocyte counts (cells/μL) in cases and controls in the different categories. **Abbreviations**: BMI, body mass index; CI, confidence interval; HCV, hepatitis C virus; IDU, intravenous drug use; MSM, men who have sex with men.

Participants in the lowest leukocyte quintile (leukocytes ≤4250/μL) and in the highest leukocyte quintile (leukocytes ≥7730/μL) were more likely to die compared to participants in the middle (reference) quintile, suggesting a U-shaped relation between leukocytes and death (**Fig 2**, **S1 Table**). OR for death (95%CI) in the lowest vs third leukocyte quintile was 2.45 (1.98–3.04). OR (95%CI) in the highest vs third leukocyte quintile was 1.82 (1.48–2.24). For comparison, univariable OR (95%CI) for hypertension, diabetes, and current smoking were 1.32 (1.15–1.52), 1.58 (1.24–2.01) and 3.48 (2.89–4.19), respectively. ORs for all leukocyte quintiles and for individual risk factors are shown in **Fig 3** and **Table 1**.

### Leukocyte count and death, multivariable model

In the final multivariable model, low and high leukocyte quintiles were significantly associated with death. Adjusted OR (95%CI) in the lowest vs. third leukocyte quintile was 1.51 (1.14–2.01). Adjusted OR (95%CI) in the highest vs. third leukocyte quintile was 1.56 (1.20–2.02) (**Figs 2 and 3**, **Table 1**). For comparison, adjusted OR (95%CI) for hypertension, diabetes, and current smoking were 1.27 (1.06–1.53), 1.91 (1.41–2.57) and 2.72 (2.14–3.45). (**Fig 3**, **Table 1**).

### Leukocyte count and death, potential confounders

Results of all bivariable analyses (leukocytes plus individual confounder variable in the analysis) are shown in **S2 Table**. These revealed a number of interactions, which were mostly discarded, however, due to lack of clinical significance (i.e., only minimally altered leukocyte-mortality ORs; **S2 Table**). Potentially relevant interactions were as follows: The effect of lowest leukocyte quintile on mortality was considerably attenuated in bivariable analysis when adjusting for CD4-count category (OR [95%CI], 1.36 [1.06–1.73]) (**S2 Table**). The effect size of highest leukocyte quintile on mortality was attenuated in bivariable analysis when adjusting for smoking (OR [95%CI], 1.45 [1.16–1.81]), consistent with smoking being associated with increased blood leukocytes [25,38–40]. Because of significant collinearity of injection drug use (IDU) and smoking, we separately adjusted for IDU, with minimal attenuation of the effect of high and low leukocytes on death (**S2 Table**, **S2A Table**).

### Sensitivity analyses

The effect of high leukocytes on mortality persisted in all sensitivity analyses. Higher ORs were noted in multivariable analysis without smoking (n = 3700; **S3 Table**), consistent with smoking being associated with increased blood leukocytes [25,38–40]. Similarly, higher ORs were noted in multivariable analysis without without obesity (n = 3144; **S4 Table**) consistent with obesity being associated with increased blood leukocytes. ORs were essentially unchanged when we analyzed only participants without leukocytosis (n = 3450; **S5 Table**). Higher ORs were noted when including only participants with suppressed viremia (n = 2140; **S6 Table**), with dyslipidemia and alcohol intake in the model (n = 1038; **S7 Table**), and in the latest sample before matching time point (n = 2894; **S8 Table**). When we added clinically indicated leukocyte measurements to the model, in addition to per protocol leukocyte measurements, ORs remained similar (n = 3700; **S9 Table**).

### Leukocytes and causes of death

**Fig 4:**
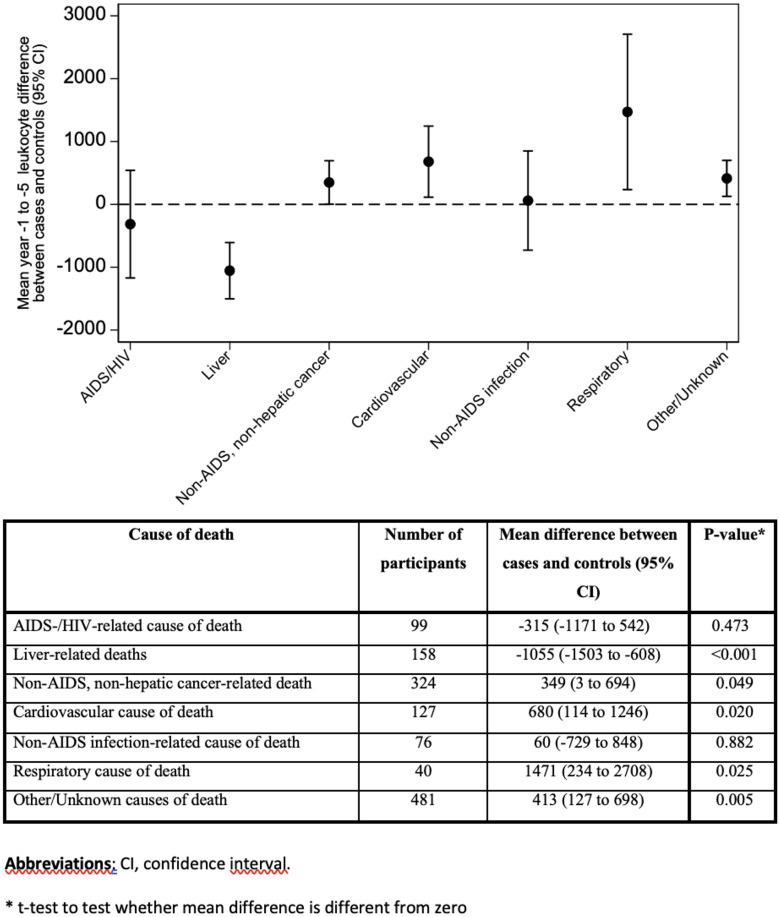
Blood Leukocytes, Measured 1 to 5 years before Death. **Mean Difference Between Cases and Controls with 95% Confidence Intervals, Stratified by Causes of Death.** Mean leukocyte difference from zero between cases and controls with 95% confidence interval for 7 groups of causes of death are shown. These values, p-values, plus number of participants in each group are tabulated below the figure. Leukocytes were significantly lower in people with HIV dying from liver-related causes compared to controls. Leukocytes were significantly higher in people with HIV dying from non-AIDS, non-hepatic cancer, cardiovascular, respiratory or other/unknown causes of death. **Abbreviations**: CI, confidence interval.

**Fig 4** shows the mean difference (95%CI) in leukocyte count >1 year before death between cases and controls, stratified by 7 major causes of death categories. Participants dying from liver-related causes had significantly lower leukocytes than controls. Participants dying from non-AIDS, non-hepatic cancer, cardiovascular causes, and respiratory causes had significantly higher leukocytes than controls, as did participants dying from other or unknown causes.

## Discussion

Our study has five main findings: First, in the final multivariable model participants in the lowest (leukocytes ≤4250/μL) and highest (leukocytes ≥7730/μL) leukocyte quintiles had significantly increased mortality compared to participants in the middle leukocyte quintile (reference; leukocyte 5290–6260/μL). This suggests a U-shaped leukocyte-mortality association, as has been previously reported in the general population [25,27]. Second, similar to the general population [25–27], elevated leukocytes, mainly within the normal range, predicted death and overt leukocytosis was uncommon. Third, the effect of high leukocytes on death persisted but was attenuated when considering smoking. This suggests that smoking, which has a well recorded association with increased leukocytes [25,38], only partially explains our observed association of leukocytes with death. Fourth, the association of low leukocytes with death (mainly from AIDS-related causes) was to a significant extent explained by low CD4 count, as might be expected, as shown by the attenuation of the effect of low leukocytes in the final multivariable model and in bivariable analysis including CD4 count. Fifth, low leukocytes were associated with liver-related causes of death, while high leukocytes were associated with death from non-AIDS non-hepatic cancer, cardiovascular and respiratory causes, consistent with findings in the general population [44–47].

In this study, we exploited the rich database of the large, well-established SHCS, which includes prospective, longitudinal data collection at 6-monthly intervals. This allowed us to analyze deaths that occurred in people with HIV in Switzerland over a 24-year period. Our finding of an independent association of high leukocytes with mortality in people with HIV appears robust, as it persisted after carefully adjusting for major traditional and HIV-associated mortality risk factors, after considering potential confounders of high leukocytes (e.g., smoking, obesity) and in several sensitivity analyses.

Our findings extend existing knowledge how chronic inflammation is involved in the pathogenesis of important non-communicable conditions and death, both in the general population and in people with HIV [8,48–50]. A number of mechanisms have been suggested that might pathophysiologically link leukocytosis (within normal range values) to increased mortality. These may include the increased production of reactive oxygen species (ROS; free oxygen radicals) by activated leukocytes [25,51-52]. ROS-mediated damage to vascular endothelium [25,51], ROS-mediated damage to pulmonary parenchymal tissue in COPD [53], increased activation, i.e., increased ROS production in neutrophils of smokers with leukocytosis [54,55], accelerated atherogenesis in patients with leukocytosis [51,40,56], an epidemiological link between leukocytosis and chronic kidney disease [57,58] and diabetes mellitus in the general population [24,59–61], and with diabetes in people with HIV [23]. Interestingly, chronic inflammation in the setting of untreated HIV Infection and residual inflammation despite successful ART has been linked to accelerated epigenetic aging in people with HIV, and in a recent large general population study, elevated leukocytes and elevated neutrophils were associated with accelerated epigenetic aging [62].

As in our previous analyses of high leukocytes predicting coronary events and diabetes in Swiss people with HIV [11,23], the effect of leukocytes on mortality was in part explained by smoking, an important risk factor for death that is well known to increase leukocytes [25,38–40]. On the other hand, while a link between overweight/obesity and increased leukocytes is well recorded [41,42], overweight/obesity did not explain the association of high leukocytes and mortality in our study of people with HIV. Indeed, obesity was associated with lower mortality in our study, consistent with findings in the general population [63].

Leukocytes represent a simple, inexpensive biomarker of inflammation and infection that is already measured routinely in clinical HIV practice. Our findings suggest how high-normal leukocytes may warrant further clinical investigation, i.e., high leukocytes should motivate HIV-clinicians to search for a potential explanation, and may suggest potential clinical interest in targeting inflammation-dampening measures in people with HIV to those with high-normal leukocytes, e.g., statins for primary coronary prevention [64]. However, formal documentation of the clinical value of routinely monitoring leukocytes would require a prospective trial, which was beyond the scope of the present study.

Our study has limitations. Our population was predominantly male, middle aged and white; results should be cautiously extrapolated to other people with HIV. We did not match cases and controls on sex. Because we required participants to contribute at least 1 year of observation time for data quality reasons, this excluded participants who died within their first year of follow-up; nonetheless, we do not believe that our results are affected by immortal time bias in any substantial fashion [65]. Possible effects of corticosteroid use, chronic inflammatory conditions other than HIV on leukocytes and mortality could not be analyzed, since these variables were not documented over the entire study duration in sufficient detail. The SHCS database does not include leukocyte differential counts, therefore we do not know whether leukocyte subsets (e.g., neutrophils, monocytes) contributed to mortality as in the general population [46]. In our previous report of CAD events in the SHCS, the association of total leukocytes and neutrophils with CAD events was similar [11]. Biomarkers such as CRP and IL-6 are not routinely recorded in the SHCS, and it is possible that joint consideration of leukocytes, CRP, and other biomarkers could improve the prediction of mortality. We have no knowledge of participants’ leukocyte levels prior to SHCS enrollment. Information on physical activity was only collected after 2009 and was therefore not included in our final model. We did not conduct a comparison of our findings with those of an HIV-negative control population. However, we report leukocyte effect sizes similar to those documented in studies from the general population [26,27].

In conclusion, we show a U-shaped association between total leukocyte count and risk of death in people with HIV in Switzerland. The association of low leukocytes with death was explained to a large extent by low CD4 count, as expected. However, high leukocytes, mostly within normal range values, robustly predict death in multivariable analyses and in multiple sensitivity analyses.

## Supporting information

S1 Table Univariable and multivariable odds ratios (95% Confidence intervals) for death according to leukocyte quintiles 1–5 years before death. (DOCX)

S2 Table Mortality odds ratios (95% Confidence Intervals) according to leukocyte quintiles and clinical variables 1–5 years before death, univariable and individual 1:1 bivariable analyses including potential interactions (Likelihood-ratio test). (DOCX)

S2A Table Collinearity between injection drug use and current smoking. (DOCX)

S3 Table Sensitivity analysis: Mortality odds ratio (95% confidence interval) in multivariable analysis excluding smoking variable in the model (n = 3700). (DOCX)

S4 Table Sensitivity analysis: Mortality odds ratio (95% Confidence Interval) in multivariable analysis excluding obese participants (n = 3144). (DOCX)

S5 Table Sensitivity analysis: Mortality odds ratio (95% Confidence Interval) in multivariable analysis excluding participants with leukocytosis (n = 3450). (DOCX)

S6 Table Sensitivity analysis: Mortality odds ratio (95% Confidence Interval) in multivariable analysis including only participants with suppressed HIV-RNA (n = 2140). (DOCX)

S7 Table Sensitivity analysis: Mortality odds ratio (95% Confidence Interval) in multivariable analysis including dyslipidemia and alcohol intake in the model (n = 1038). (DOCX)

S8 Table Sensitivity analysis: Mortality odds ratio (95% Confidence Interval) in multivariable analysis at latest available time point before death (n = 2894). (DOCX)

S9 Table Sensitivity analysis: Mortality Odds Ratio (95% Confidence Interval) in multivariable analysis including clinically indicated leukocyte measurements in addition to per protocol leukocyte measurements (n = 3700). (DOCX)
