# Supplementary material for: Association of leukocyte count with death in people with HIV: A longitudinal study over 24 years
Source: PLoS One. 2026 Jan 8;21(1):e0340678. doi: 10.1371/journal.pone.0340678 (PMC12782362; doi:10.1371/journal.pone.0340678)
Supplement: S1 Table — (DOCX) [file pone.0340678.s001.docx]

**S1 Table: Univariable and Multivariable Odds Ratios (95% Confidence Intervals) for Death According to Leukocyte Quintiles 1 to 5 Years before Death**

|  | **Leukocyte Range (cells/μL)** | **Univariable Analysis** | **Multivariable Analysis** |
| --- | --- | --- | --- |
| 1^st^ (lowest) leukocyte quintile | 6-4250 | 2.45 (1.98-3.04); p<0.001 | 1.51 (1.14-2.01); p=0.004 |
| 2nd leukocyte quintile | 4270-5280 | 1.24 (1.01-1.53); p=0.041 | 1.11 (0.86-1.45); p=0.421 |
| 3rd leukocyte quintile | 5290-6260 | (reference) | (reference) |
| 4th leukocyte quintile | 6270-7720 | 1.18 (0.96-1.45); p=0.124 | 1.09 (0.84-1.42); p=0.522 |
| 5th (highest) leukocyte quintile | >7730 | 1.82 (1.48-2.24); p<0.001 | 1.56 (1.20-2.02); p=0.001 |
