# Supplementary material for: Association of leukocyte count with death in people with HIV: A longitudinal study over 24 years
Source: PLoS One. 2026 Jan 8;21(1):e0340678. doi: 10.1371/journal.pone.0340678 (PMC12782362; doi:10.1371/journal.pone.0340678)
Supplement: S2 Table — (DOCX) [file pone.0340678.s002.docx]

**S2 Table: Mortality Odds Ratios (95% Confidence Intervals) According to Leukocyte Quintiles and Clinical Variables 1 to 5 Years before Death, Univariable and Individual 1:1 Bivariable Analyses Including Potential Interactions (Likelihood-Ratio Test)**

|  | **Univariable Analysis** | **Bivariable Analysis** | **Likelihood-Ratio Test for Interaction** |
| --- | --- | --- | --- |
| **Sex:** male | (reference) | (reference) | P=0.559 |
| **Sex:** female | 1.00 (0.86-1.17); p=0.968 | 0.96 (0.82-1.13); p=0.611 |  |
| 1^st^ (lowest) leukocyte quintile | *2.45 (1.98-3.04); p<0.001* | 2.46 (1.99-3.06); p<0.001 |  |
| 2nd leukocyte quintile | *1.24 (1.01-1.53); p=0.041* | 1.24 (1.01-1.53); p=0.039 |  |
| 3rd leukocyte quintile | *(reference)* | (reference) |  |
| 4th leukocyte quintile | *1.18 (0.96-1.45); p=0.124* | 1.18 (0.96-1.45); p=0.120 |  |
| 5th (highest) leukocyte quintile | *1.82 (1.48-2.24); p<0.001* | 1.82 (1.48-2.24); p<0.001 |  |
| **Ethnicity:** White | (reference) | (reference) | P=0.052 |
| **Ethnicity:** Black | 1.20 (0.86-1.67); p=0.294 | 1.06 (0.75-1.50); p=0.726 |  |
| **Ethnicity:** Hispanic | 0.41 (0.22-0.77); p=0.006 | 0.35 (0.18-0.67); p=0.001 |  |
| **Ethnicity:** Asian | 0.45 (0.26-0.79); p= 0.005 | 0.49 (0.28-0.86); p=0.012 |  |
| 1^st^ (lowest) leukocyte quintile | *2.45 (1.98-3.04); p<0.001* | 2.50 (2.01-3.10); p<0.001 |  |
| 2nd leukocyte quintile | *1.24 (1.01-1.53); p=0.041* | 1.25 (1.02-1.55); p=0.033 |  |
| 3rd leukocyte quintile | *(reference)* | (reference) |  |
| 4th leukocyte quintile | *1.18 (0.96-1.45); p=0.124* | 1.18 (0.96-1.46); p=0.120 |  |
| 5th (highest) leukocyte quintile | *1.82 (1.48-2.24); p<0.001* | 1.80 (1.46-2.22); p<0.001 |  |
| **Risk Group:** MSM | (reference) | (reference) | P=0.011 |
| **Risk Group:** IDU | 4.47 (3.64-5.49); p<0.001 | 4.29 (3.48-5.29); p<0.001 |  |
| **Risk Group:** heterosexual | 1.61 (1.36-1.92); p<0.001 | 1.55 (1.30-1.85); p<0.001 |  |
| **Risk Group:** other | 1.57 (1.06-2.31); p=0.024 | 1.52 (1.02-2.27); p=0.038 |  |
| 1^st^ (lowest) leukocyte quintile | *2.45 (1.98-3.04); p<0.001* | 2.25 (1.79-2.82); p<0.001 |  |
| 2nd leukocyte quintile | *1.24 (1.01-1.53); p=0.041* | 1.16 (0.93-1.44); p=0.190 |  |
| 3rd leukocyte quintile | *(reference)* | (reference) |  |
| 4th leukocyte quintile | *1.18 (0.96-1.45); p=0.124* | 1.12 (0.90-1.40); p=0.319 |  |
| 5th (highest) leukocyte quintile | *1.82 (1.48-2.24); p<0.001* | 1.64 (1.32-2.05); p<0.001 |  |
| **Risk Group: Injection drug use** | 3.59 (2.98-4.33); p<0.001 | 3.49 (2.89-4.23); p<0.001 | P=0.017 |
| 1^st^ (lowest) leukocyte quintile | *2.45 (1.98-3.04); p<0.001* | 2.34 (1.86-2.93); p<0.001 |  |
| 2nd leukocyte quintile | *1.24 (1.01-1.53); p=0.041* | 1.17 (0.94-1.46); p=0.152 |  |
| 3rd leukocyte quintile | *(reference)* | (reference) |  |
| 4th leukocyte quintile | *1.18 (0.96-1.45); p=0.124* | 1.16 (0.93-1.45); p=0.175 |  |
| 5th (highest) leukocyte quintile | *1.82 (1.48-2.24); p<0.001* | 1.69 (1.36-2.10); p<0.001 |  |
| **Smoking:** Never | (reference) | (reference) | P<0.01 |
| **Smoking:** Current smoking | 3.48 (2.89-4.19); p<0.001 | 3.80 (3.13-4.63); p<0.001 |  |
| **Smoking:** Past smoking | 1.55 (1.27-1.88); p<0.001 | 1.55 (1.26-1.90); p<0.001 |  |
| 1^st^ (lowest) leukocyte quintile | *2.45 (1.98-3.04); p<0.001* | 2.71 (2.15-3.41); p<0.001 |  |
| 2^nd^ leukocyte quintile | *1.24 (1.01-1.53); p=0.041* | 1.30 (1.04-1.62); p=0.020 |  |
| 3rd leukocyte quintile | *(reference)* | (reference) |  |
| 4th leukocyte quintile | *1.18 (0.96-1.45); p=0.124* | 0.98 (0.79-1.23); p=0.882 |  |
| 5th (highest) leukocyte quintile | *1.82 (1.48-2.24); p<0.001* | 1.45 (1.16-1.81); p=0.001 |  |
| **Education:** Mandatory School | (reference) | (reference) | P=0.311 |
| **Education:** Apprenticeship | 0.59 (0.50-0.70); p<0.001 | 0.60 (0.50-0.71); p<0.001 |  |
| **Education:** Higher Education | 0.43 (0.35-0.53); p<0.001 | 0.44 (0.36-0.55); p<0.001 |  |
| **Education:** Other/Missing | 0.78 (0.58-1.06); p=0.111 | 0.79 (0.58-1.08); p=0.144 |  |
| 1^st^ (lowest) leukocyte quintile | *2.45 (1.98-3.04); p<0.001* | 2.42 (1.95-3.01); p<0.001 |  |
| 2nd leukocyte quintile | *1.24 (1.01-1.53); p=0.041* | 1.25 (1.01-1.54); p=0.041 |  |
| 3rd leukocyte quintile | *(reference)* | (reference) |  |
| 4th leukocyte quintile | *1.18 (0.96-1.45); p=0.124* | 1.15 (0.93-1.43); p=0.187 |  |
| 5th (highest) leukocyte quintile | *1.82 (1.48-2.24); p<0.001* | 1.75 (1.42-2.16); p<0.001 |  |
| **BMI** Underweight | 4.33 (3.18-5.89); p<0.001 | 4.23 (3.09-5.78); p<0.001 | P=0.061 |
| **BMI**: Normal | (reference) | (reference) |  |
| **BMI**: Overweight | 0.70 (0.59-0.82); p<0.001 | 0.72 (0.61-0.84); p<0.001 |  |
| **BMI**: Obese | 0.81 (0.63-1.05); p=0.107 | 0.86 (0.67-1.12); p=0.266 |  |
| 1st (lowest) leukocyte quintile | *2.45 (1.98-3.04); p<0.001* | 2.35 (1.88-2.94); p<0.001 |  |
| 2nd leukocyte quintile | *1.24 (1.01-1.53); p=0.041* | 1.26 (1.02-1.57); p=0.032 |  |
| 3rd leukocyte quintile | *(reference)* | (reference) |  |
| 4th leukocyte quintile | *1.18 (0.96-1.45); p=0.124* | 1.19 (0.96-1.48); p=0.115 |  |
| 5th (highest) leukocyte quintile | *1.82 (1.48-2.24); p<0.001* | 1.81 (1.46-2.24); p<0.001 |  |
| **Hypertension** | 1.32 (1.15-1.52); p<0.001 | 1.34 (1.16-1.54); p<0.001 | P=0.992 |
| 1st (lowest) leukocyte quintile | *2.45 (1.98-3.04); p<0.001* | 2.47 (1.99-3.06); p<0.001 |  |
| 2nd leukocyte quintile | *1.24 (1.01-1.53); p=0.041* | 1.24 (1.01-1.53); p=0.041 |  |
| 3rd leukocyte quintile | *(reference)* | (reference) |  |
| 4th leukocyte quintile | *1.18 (0.96-1.45); p=0.124* | 1.18 (0.95-1.45); p=0.130 |  |
| 5th (highest) leukocyte quintile | *1.82 (1.48-2.24); p<0.001* | 1.83 (1.48-2.25); p<0.001 |  |
| **CMV seropositivity** | 0.75 (0.63-0.89); p=0.001 | 0.78 (0.66-0.94); p=0.009 | P=0.876 |
| 1st (lowest) leukocyte quintile | *2.45 (1.98-3.04); p<0.001* | 2.42 (1.95-3.01); p<0.001 |  |
| 2nd leukocyte quintile | *1.24 (1.01-1.53); p=0.041* | 1.24 (1.01-1.52); p=0.043 |  |
| 3rd leukocyte quintile | *(reference)* | (reference) |  |
| 4th leukocyte quintile | *1.18 (0.96-1.45); p=0.124* | 1.17 (0.95-1.44); p=0.137 |  |
| 5th (highest) leukocyte quintile | *1.82 (1.48-2.24); p<0.001* | 1.79 (1.46-2.21); p<0.001 |  |
| **Hepatitis C seropositivity** | 3.29 (2.76-3.91); p<0.001 | 3.17 (2.65-3.78); p<0.001 | P=0.058 |
| 1st (lowest) leukocyte quintile | *2.45 (1.98-3.04); p<0.001* | 2.30 (1.83-2.88); p<0.001 |  |
| 2nd leukocyte quintile | *1.24 (1.01-1.53); p=0.041* | 1.24 (0.99-1.54); p=0.057 |  |
| 3rd leukocyte quintile | *(reference)* | (reference) |  |
| 4th leukocyte quintile | *1.18 (0.96-1.45); p=0.124* | 1.17 (0.94-1.46); p= 0.150 |  |
| 5th (highest) leukocyte quintile | *1.82 (1.48-2.24); p<0.001* | 1.75 (1.41-2.17); p<0.001 |  |
| **Diabetes** | 1.58 (1.24-2.01); p<0.001 | 1.63 (1.27-2.09); p<0.001 | P=0.249 |
| 1st (lowest) leukocyte quintile | *2.45 (1.98-3.04); p<0.001* | 2.50 (2.01-3.10); p<0.001 |  |
| 2nd leukocyte quintile | *1.24 (1.01-1.53); p=0.041* | 1.27 (1.03-1.56); p=0.024 |  |
| 3rd leukocyte quintile | *(reference)* | (reference) |  |
| 4th leukocyte quintile | *1.18 (0.96-1.45); p=0.124* | 1.19 (0.96-1.46); p=0.112 |  |
| 5th (highest) leukocyte quintile | *1.82 (1.48-2.24); p<0.001* | 1.83 (1.49-2.26); p<0.001 |  |
| **Family History of CAD** | 0.97 (0.79-1.19); p=0.756 | 0.98 (0.80-1.21); p=0.864 | P=0.437 |
| 1st (lowest) leukocyte quintile | *2.45 (1.98-3.04); p<0.001* | 2.45 (1.98-3.04); p<0.001 |  |
| 2nd leukocyte quintile | *1.24 (1.01-1.53); p=0.041* | 1.24 (1.01-1.53); p=0.041 |  |
| 3rd leukocyte quintile | *(reference)* | (reference) |  |
| 4th leukocyte quintile | *1.18 (0.96-1.45); p=0.124* | 1.18 (0.96-1.45); p=0.123 |  |
| 5th (highest) leukocyte quintile | *1.82 (1.48-2.24); p<0.001* | 1.82 (1.48-2.24); p<0.001 |  |
| **CD4 nadir <50cells/ μL** | 1.61 (1.38-1.88); p<0.001 | 1.51 (1.29-1.77); p<0.001 | P=0.004 |
| 1st (lowest) leukocyte quintile | *2.45 (1.98-3.04); p<0.001* | 2.31 (1.86-2.87); p<0.001 |  |
| 2nd leukocyte quintile | *1.24 (1.01-1.53); p=0.041* | 1.23 (1.00-1.52); p=0.051 |  |
| 3rd leukocyte quintile | *(reference)* | (reference) |  |
| 4th leukocyte quintile | *1.18 (0.96-1.45); p=0.124* | 1.18 (0.96-1.46); p=0.124 |  |
| 5th (highest) leukocyte quintile | *1.82 (1.48-2.24); p<0.001* | 1.83 (1.49-2.26); p<0.001 |  |
| **HIV RNA status, <50 copies/mL (undetectable)** | 0.43 (0.36-0.52); p<0.001 | 0.44 (0.37-0.53); p<0.001 | P=0.261 |
| 1st (lowest) leukocyte quintile | *2.45 (1.98-3.04); p<0.001* | 2.34 (1.88-2.92); p<0.001 |  |
| 2nd leukocyte quintile | *1.24 (1.01-1.53); p=0.041* | 1.19 (0.97-1.48), p=0.100 |  |
| 3rd leukocyte quintile | *(reference)* | (reference) |  |
| 4th leukocyte quintile | *1.18 (0.96-1.45); p=0.124* | 1.19 (0.96-1.47); p=0.117 |  |
| 5th (highest) leukocyte quintile | *1.82 (1.48-2.24); p<0.001* | 1.84 (1.49-2.27); p<0.001 |  |
| **CD4 cell count category at matching date**: <200 | (reference) | (reference) | P=0.122 |
| **CD4 cell count category at matching date**: 200-<350 cells/μL | 0.37 (0.28-0.49); p<0.001 | 0.38 (0.29-0.51); p<0.001 |  |
| **CD4 cell count category at matching date**: 350-<500 cells/μL | 0.18 (0.14-0.24); p<0.001 | 0.18 (0.14-0.24); p<0.001 |  |
| **CD4 cell count category at matching date**: ≥500 cells/μL | 0.13 (0.10-0.17); p<0.001 | 0.12 (0.09-0.16); p<0.001 |  |
| 1st (lowest) leukocyte quintile | *2.45 (1.98-3.04); p<0.001* | 1.36 (1.06-1.73); p=0.014 |  |
| 2nd leukocyte quintile | *1.24 (1.01-1.53); p=0.041* | 0.98 (0.78-1.22); p=0.828 |  |
| 3rd leukocyte quintile | *(reference)* | (reference) |  |
| 4th leukocyte quintile | *1.18 (0.96-1.45); p=0.124* | 1.27 (1.02-1.60); p=0.035 |  |
| 5th (highest) leukocyte quintile | *1.82 (1.48-2.24); p<0.001* | 2.08 (1.66-2.61); p<0.001 |  |

**Abbreviations.** BMI, body mass index; CAD, coronary artery disease, CMV, cytomegalovirus; HCV, Hepatitis C Virus; IDU, injection drug use
