# Supplementary material for: Association of leukocyte count with death in people with HIV: A longitudinal study over 24 years
Source: PLoS One. 2026 Jan 8;21(1):e0340678. doi: 10.1371/journal.pone.0340678 (PMC12782362; doi:10.1371/journal.pone.0340678)
Supplement: S2A Table — (DOCX) [file pone.0340678.s003.docx]

**S2A Table: Collinearity between Injection Drug Use and Current Smoking**

|  | **Smoking status** | | |  |
| --- | --- | --- | --- | --- |
|  | **Never** | **Current** | **Past** | **Total** |
| **No injection drug use** | 1064 (40.3) | 889 (33.7) | 686 (26.0) | 2639 (100.0) |
| **Injection drug use** | 196 (18.5) | 704 (66.4) | 161 (15.2) | 1061 (100.0) |
| **Total** | 1260 (34.1) | 1593 (43.1) | 847 (22.9) | 3700 (100.0) |
|  | Fisher’s exact test: p<0.001 | | | |

**Note.** All data shown apply to the matching date and are number (%) of participants, unless otherwise indicated.
