# Supplementary material for: Association of leukocyte count with death in people with HIV: A longitudinal study over 24 years
Source: PLoS One. 2026 Jan 8;21(1):e0340678. doi: 10.1371/journal.pone.0340678 (PMC12782362; doi:10.1371/journal.pone.0340678)
Supplement: S3 Table — (DOCX) [file pone.0340678.s004.docx]

**S3 Table: Sensitivity Analysis: Mortality Odds Ratio (95% Confidence Interval) in Multivariable Analysis Excluding Smoking Variable in the Model (n=3700)**

|  | **Multivariable analysis** |
| --- | --- |
| 1^st^ (lowest) leukocyte quintile* | 1.37 (1.04-1.80); p=0.025 |
| 2nd leukocyte quintile* | 1.06 (0.82-1.37); p=0.640 |
| 3rd leukocyte quintile* | (reference) |
| 4th leukocyte quintile* | 1.24 (0.96-1.60); p=0.092 |
| 5th (highest) leukocyte quintile* | 1.78 (1.39-2.29); p<0.001 |
| **Sex:** male | (reference) |
| **Sex:** female | 0.63 (0.50-0.80); p<0.001 |
| **Ethnicity:** White | (reference) |
| **Ethnicity:** Black | 1.22 (0.78-1.90); p=0.388 |
| **Ethnicity:** Hispanic | 0.39 (0.18-0.85); p=0.017 |
| **Ethnicity:** Asian | 0.45 (0.23-0.89); p=0.021 |
| **HIV acquisition mode:** MSM | (reference) |
| **HIV acquisition mode:** IDU | 2.50 (1.80-3.48); p<0.001 |
| **HIV acquisition mode:** Heterosexual | 1.61 (1.26-2.06); p<0.001 |
| **HIV acquisition mode:** Other | 1.18 (0.74-1.87); p=0.492 |
| **Education:** Mandatory School | (reference) |
| **Education:** Apprenticeship | 0.69 (0.55-0.86); p=0.001 |
| **Education:** Higher Education | 0.63 (0.48-0.82); p=0.001 |
| **Education:** Other/Missing | 1.18 (0.74-1.87); p=0.492 |
| **BMI:** Underweight | 3.77 (2.61-5.44); p<0.001 |
| **BMI:** Normal | (reference) |
| **BMI:** Overweight | 0.72 (0.59-0.87); p=0.001 |
| **BMI:** Obese | 0.68 (0.51-0.92); p=0.012 |
| **Hypertension** | 1.29 (1.08-1.54); p=0.004 |
| **Hepatitis C seropositivity** | 1.66 (1.27-2.18); p<0.001 |
| **Diabetes** | 1.81 (1.36-2.42); p<0.001 |
| **HIV RNA <50 copies/mL** | 0.51 (0.41-0.64); p<0.001 |

**Abbreviations.** BMI, body mass index; IDU, injection drug use; MSM, men who have sex with men

* leukocyte count 1 to 5 years before matching date
