# Supplementary material for: Association of leukocyte count with death in people with HIV: A longitudinal study over 24 years
Source: PLoS One. 2026 Jan 8;21(1):e0340678. doi: 10.1371/journal.pone.0340678 (PMC12782362; doi:10.1371/journal.pone.0340678)
Supplement: S4 Table — (DOCX) [file pone.0340678.s005.docx]

**S4 Table: Sensitivity Analysis: Mortality Odds Ratio (95% Confidence Interval) in Multivariable Analysis Excluding Obese Participants (n=3144)**

|  | **Multivariable analysis** |
| --- | --- |
| 1^st^ (lowest) leukocyte quintile* | 1.74 (1.28-2.37); p<0.001 |
| 2nd leukocyte quintile* | 1.19 (0.89-1.58); p=0.240 |
| 3rd leukocyte quintile* | (reference) |
| 4th leukocyte quintile* | 1.19 (0.89-1.59); p=0.247 |
| 5th (highest) leukocyte quintile* | 1.61 (1.21-2.14); p=0.001 |
| **Sex:** male | (reference) |
| **Sex:** female | 0.66 (0.51-0.86); p=0.002 |
| **Ethnicity:** White | (reference) |
| **Ethnicity:** Black | 1.63 (0.94-2.82); p=0.080 |
| **Ethnicity:** Hispanic | 0.39 (0.16-0.95); p=0.038 |
| **Ethnicity:** Asian | 0.61 (0.28-1.31); p=0.205 |
| **HIV acquisition mode:** MSM | (reference) |
| **HIV acquisition mode:** IDU | 2.28 (1.59-3.29); p<0.001 |
| **HIV acquisition mode:** Heterosexual | 1.67 (1.26-2.21); p<0.001 |
| **HIV acquisition mode:** Other | 1.42 (0.84-2.42); p=0.191 |
| **Smoking:** never | (reference) |
| **Smoking:** current smoking | 2.80 (2.14-3.66); p<0.001 |
| **Smoking:** past smoking | 1.46 (1.11-1.93); p=0.007 |
| **Education:** Mandatory School | (reference) |
| **Education:** Apprenticeship | 0.66 (0.51-0.85); p=0.001 |
| **Education:** Higher Education | 0.65 (0.48-0.88); p=0.005 |
| **Education:** Other/Missing | 0.66 (0.44-1.00); p=0.049 |
| **BMI:** Underweight | 3.40 (2.31-5.00); p<0.001 |
| **BMI:** Normal | (reference) |
| **BMI:** Overweight | 0.77 (0.63-0.96); p=0.018 |
| **Hypertension** | 1.29 (1.05-1.58); p=0.013 |
| **Hepatitis C seropositivity** | 1.47 (1.09-1.99); p=0.012 |
| **Diabetes** | 1.94 (1.37-2.74); p<0.001 |
| **HIV RNA <50 copies/mL** | 0.47 (0.36-0.60); p<0.001 |

**Abbreviations.** BMI, body mass index; IDU, injection drug use; MSM, men who have sex with men

* leukocyte count 1 to 5 years before matching date
