# Supplementary material for: Association of leukocyte count with death in people with HIV: A longitudinal study over 24 years
Source: PLoS One. 2026 Jan 8;21(1):e0340678. doi: 10.1371/journal.pone.0340678 (PMC12782362; doi:10.1371/journal.pone.0340678)
Supplement: S5 Table — (DOCX) [file pone.0340678.s006.docx]

**S5 Table: Sensitivity Analysis: Mortality Odds Ratio (95% Confidence Interval) in Multivariable Analysis Excluding Participants with Leukocytosis (n=3450)**

|  | **Multivariable analysis** |
| --- | --- |
| 1^st^ (lowest) leukocyte quintile* | 1.50 (1.12-2.00); p=0.006 |
| 2nd leukocyte quintile* | 1.09 (0.83-1.42); p=0.531 |
| 3rd leukocyte quintile* | (reference) |
| 4th leukocyte quintile* | 1.08 (0.83-1.41); p=0.573 |
| 5th (highest) leukocyte quintile* | 1.44 (1.09-1.89); p=0.010 |
| **Sex:** male | (reference) |
| **Sex:** female | 0.61 (0.48-0.79); p<0.001 |
| **Ethnicity:** White | (reference) |
| **Ethnicity:** Black | 1.57 (0.97-2.53); p=0.064 |
| **Ethnicity:** Hispanic | 0.40 (0.17-0.91); p=0.029 |
| **Ethnicity:** Asian | 0.55 (0.26-1.12); p=0.101 |
| **HIV acquisition mode:** MSM | (reference) |
| **HIV acquisition mode:** IDU | 1.86 (1.31-2.65); p=0.001 |
| **HIV acquisition mode:** Heterosexual | 1.57 (1.21-2.04); p=0.001 |
| **HIV acquisition mode:** Other | 1.31 (0.80-2.16); p=0.286 |
| **Smoking:** never | (reference) |
| **Smoking:** current smoking | 2.83 (2.21-3.62); p<0.001 |
| **Smoking:** past smoking | 1.39 (1.08-1.78); p=0.009 |
| **Education:** Mandatory School | (reference) |
| **Education:** Apprenticeship | 0.74 (0.58-0.93); p=0.012 |
| **Education:** Higher Education | 0.71 (0.54-0.95); p=0.019 |
| **Education:** Other/Missing | 0.80 (0.54-1.18); p=0.264 |
| **BMI:** Underweight | 3.62 (2.45-5.36); p<0.001 |
| **BMI:** Normal | (reference) |
| **BMI:** Overweight | 0.79 (0.64-0.97); p=0.026 |
| **BMI:** Obese | 0.79 (0.58-1.09); p=0.155 |
| **Hypertension** | 1.23 (1.02-1.49); p=0.032 |
| **Hepatitis C seropositivity** | 1.64 (1.22-2.19); p=0.001 |
| **Diabetes** | 1.94 (1.42-2.66); p<0.001 |
| **HIV RNA <50 copies/mL** | 0.55 (0.44-0.70); p<0.001 |

**Abbreviations.** BMI, body mass index; IDU, injection drug use; MSM, men who have sex with men

* leukocyte count 1 to 5 years before matching date
