# Supplementary material for: Association of leukocyte count with death in people with HIV: A longitudinal study over 24 years
Source: PLoS One. 2026 Jan 8;21(1):e0340678. doi: 10.1371/journal.pone.0340678 (PMC12782362; doi:10.1371/journal.pone.0340678)
Supplement: S6 Table — (DOCX) [file pone.0340678.s007.docx]

**S6 Table: Sensitivity Analysis: Mortality Odds Ratio (95% Confidence Interval) in Multivariable Analysis Including Only Participants with Suppressed HIV-RNA (n=2140)**

|  | **Multivariable analysis** |
| --- | --- |
| 1^st^ (lowest) leukocyte quintile* | 1.55 (1.06-2.27); p=0.023 |
| 2nd leukocyte quintile* | 1.36 (0.97-1.91); p=0.076 |
| 3rd leukocyte quintile* | (reference) |
| 4th leukocyte quintile* | 1.07 (0.77-1.50); p=0.672 |
| 5th (highest) leukocyte quintile* | 1.82 (1.32-2.52); p<0.001 |
| **Sex:** male | (reference) |
| **Sex:** female | 0.55 (0.40-0.76); p<0.001 |
| **Ethnicity:** White | (reference) |
| **Ethnicity:** Black | 1.39 (0.75-2.58); p=0.293 |
| **Ethnicity:** Hispanic | 0.28 (0.10-0.84); p=0.023 |
| **Ethnicity:** Asian | 0.83 (0.33-2.10); p=0.701 |
| **HIV acquisition mode:** MSM | (reference) |
| **HIV acquisition mode:** IDU | 2.08 (1.33-3.26); p=0.001 |
| **HIV acquisition mode:** Heterosexual | 1.79 (1.29-2.48); p=0.001 |
| **HIV acquisition mode:** Other | 1.19 (0.66-2.14); p=0.566 |
| **Smoking:** never | (reference) |
| **Smoking:** current smoking | 3.48 (2.54-4.77); p<0.001 |
| **Smoking:** past smoking | 1.50 (1.12-2.01); p=0.007 |
| **Education:** Mandatory School | (reference) |
| **Education:** Apprenticeship | 0.65 (0.48-0.88); p=0.006 |
| **Education:** Higher Education | 0.66 (0.46-0.95); p=0.024 |
| **Education:** Other/Missing | 0.68 (0.41-1.13); p=0.139 |
| **BMI:** Underweight | 3.15 (1.89-5.24); p<0.001 |
| **BMI:** Normal | (reference) |
| **BMI:** Overweight | 0.72 (0.56-0.93); p=0.013 |
| **BMI:** Obese | 0.68 (0.46-0.98); p=0.041 |
| **Hypertension** | 1.22 (0.98-1.53); p=0.080 |
| **Hepatitis C seropositivity** | 1.47 (1.02-2.12); p=0.036 |
| **Diabetes** | 1.79 (1.27-2.54); p=0.001 |

**Abbreviations.** BMI, body mass index; IDU, injection drug use; MSM, men who have sex with men

* leukocyte count 1 to 5 years before matching date
