# Supplementary material for: Association of leukocyte count with death in people with HIV: A longitudinal study over 24 years
Source: PLoS One. 2026 Jan 8;21(1):e0340678. doi: 10.1371/journal.pone.0340678 (PMC12782362; doi:10.1371/journal.pone.0340678)
Supplement: S7 Table — (DOCX) [file pone.0340678.s008.docx]

**S7 Table: Sensitivity Analysis: Mortality Odds Ratio (95% Confidence Interval) in Multivariable Analysis Including Dyslipidemia and Alcohol Intake in the Model (n=1038)**

|  | **Multivariable analysis** |
| --- | --- |
| 1^st^ (lowest) leukocyte quintile* | 1.17 (0.67-2.04); p=0.570 |
| 2nd leukocyte quintile* | 1.03 (0.64-1.66); p=0.912 |
| 3rd leukocyte quintile* | (reference) |
| 4th leukocyte quintile* | 0.89 (0.57-1.39); p=0.611 |
| 5th (highest) leukocyte quintile* | 1.68 (1.07-2.63); p=0.024 |
| **Sex:** male | (reference) |
| **Sex:** female | 0.57 (0.36-0.90); p=0.016 |
| **Ethnicity:** White | (reference) |
| **Ethnicity:** Black | 1.77 (0.77-4.07); p=0.178 |
| **Ethnicity:** Hispanic | n.c. |
| **Ethnicity:** Asian | 0.82 (0.18-3.86); p=0.806 |
| **HIV acquisition mode:** MSM | (reference) |
| **HIV acquisition mode:** IDU | 1.73 (0.92-3.26); p=0.089 |
| **HIV acquisition mode:** Heterosexual | 1.69 (1.08-2.62); p=0.021 |
| **HIV acquisition mode:** Other | 0.85 (0.36-2.03); p=0.721 |
| **Smoking:** never | (reference) |
| **Smoking:** current smoking | 3.47 (2.17-5.55); p<0.001 |
| **Smoking:** past smoking | 1.87 (1.22-2.87); p=0.004 |
| **Education:** Mandatory School | (reference) |
| **Education:** Apprenticeship | 0.76 (0.49-1.18); p=0.218 |
| **Education:** Higher Education | 0.85 (0.51-1.42); p=0.528 |
| **Education:** Other/Missing | 0.54 (0.27-1.08); p=0.081 |
| **BMI:** Underweight | 3.88 (1.79-8.39); p=0.001 |
| **BMI:** Normal | (reference) |
| **BMI:** Overweight | 0.87 (0.61-1.25); p=0.457 |
| **BMI:** Obese | 0.88 (0.51-1.50); p=0.631 |
| **Hypertension** | 1.18 (0.86-1.61); p=0.303 |
| **Hepatitis C seropositivity** | 1.54 (0.93-2.57); p=0.097 |
| **Diabetes** | 2.05 (1.29-3.26); p=0.002 |
| **HIV RNA <50 copies/mL** | 0.83 (0.44-1.56); p=0.567 |
| **Alcohol intake:** none/mild | (reference) |
| **Alcohol intake:** moderate/heavy | 0.88 (0.64-1.22); p=0.438 |
| **Dyslipidemia** | 0.80 (0.58-1.09); p=0.158 |

**Abbreviations.** BMI, body mass index; IDU, injection drug use; MSM, men who have sex with men;

n.c., not computable since no hispanic case had information on both alcohol intake and dyslipidemia

* leukocyte count 1 to 5 years before matching date
