# Supplementary material for: Association of leukocyte count with death in people with HIV: A longitudinal study over 24 years
Source: PLoS One. 2026 Jan 8;21(1):e0340678. doi: 10.1371/journal.pone.0340678 (PMC12782362; doi:10.1371/journal.pone.0340678)
Supplement: S8 Table — (DOCX) [file pone.0340678.s009.docx]

**S8 Table: Sensitivity Analysis: Mortality Odds Ratio (95% Confidence Interval) in Multivariable Analysis at Latest Available Time Point before Death (n=2894)**

|  | **Multivariable analysis** |
| --- | --- |
| 1^st^ (lowest) leukocyte quintile* | 1.29 (0.91-1.81); p=0.149 |
| 2nd leukocyte quintile* | 1.04 (0.75-1.43); p=0.823 |
| 3rd leukocyte quintile* | (reference) |
| 4th leukocyte quintile* | 1.17 (0.85-1.60); p=0.325 |
| 5th (highest) leukocyte quintile* | 1.99 (1.44-2.76); p<0.001 |
| **Sex:** male | (reference) |
| **Sex:** female | 0.60 (0.44-0.82); p=0.001 |
| **Ethnicity:** White | (reference) |
| **Ethnicity:** Black | 1.36 (0.76-2.46); p=0.303 |
| **Ethnicity:** Hispanic | 0.30 (0.10-0.88); p=0.028 |
| **Ethnicity:** Asian | 0.60 (0.26-1.39); p=0.235 |
| **HIV acquisition mode:** MSM | (reference) |
| **HIV acquisition mode:** IDU | 1.88 (1.23-2.90); p=0.004 |
| **HIV acquisition mode:** Heterosexual | 1.50 (1.10-2.04); p=0.011 |
| **HIV acquisition mode:** Other | 0.88 (0.49-1.57); p=0.667 |
| **Smoking:** never | (reference) |
| **Smoking:** current smoking | 2.51 (1.87-3.37); p<0.001 |
| **Smoking:** past smoking | 1.34 (1.01-1.77); p=0.046 |
| **Education:** Mandatory School | (reference) |
| **Education:** Apprenticeship | 0.73 (0.55-0.98); p=0.036 |
| **Education:** Higher Education | 0.84 (0.60-1.17); p=0.300 |
| **Education:** Other/Missing | 0.72 (0.45-1.15); p=0.168 |
| **BMI:** Underweight | 4.11 (2.67-6.31); p<0.001 |
| **BMI:** Normal | (reference) |
| **BMI:** Overweight | 0.65 (0.52-0.83); p=0.001 |
| **BMI:** Obese | 0.68 (0.47-0.99); p=0.046 |
| **Hypertension** | 1.42 (1.14-1.77); p=0.002 |
| **Hepatitis C seropositivity** | 1.70 (1.20-2.42); p=0.003 |
| **Diabetes** | 1.82 (1.29-2.58); p=0.001 |
| **HIV RNA <50 copies/mL** | 0.66 (0.49-0.91); p=0.010 |

**Abbreviations.** BMI, body mass index; IDU, injection drug use; MSM, men who have sex with men

* latest leukocyte count before matching date
