# Supplementary material for: Association of leukocyte count with death in people with HIV: A longitudinal study over 24 years
Source: PLoS One. 2026 Jan 8;21(1):e0340678. doi: 10.1371/journal.pone.0340678 (PMC12782362; doi:10.1371/journal.pone.0340678)
Supplement: S9 Table — (DOCX) [file pone.0340678.s010.docx]

**S9 Table: Sensitivity Analysis: Mortality Odds Ratio (95% Confidence Interval) in Multivariable Analysis Including Clinically Indicated Leukocyte Measurements in Addition to Per Protocol Leukocyte Measurements (n=3700)**

|  | **Multivariable analysis** |
| --- | --- |
| 1^st^ (lowest) leukocyte quintile* | 1.41 (1.06-1.87); p=0.018 |
| 2nd leukocyte quintile* | 1.12 (0.86-1.46); p=0.395 |
| 3rd leukocyte quintile* | (reference) |
| 4th leukocyte quintile* | 1.22 (0.94-1.58); p=0.142 |
| 5th (highest) leukocyte quintile* | 1.57 (1.21-2.04); p=0.001 |
| **Sex:** male | (reference) |
| **Sex:** female | 0.63 (0.50-0.80); p<0.001 |
| **Ethnicity:** White | (reference) |
| **Ethnicity:** Black | 1.51 (0.95-2.41); p=0.080 |
| **Ethnicity:** Hispanic | 0.36 (0.16-0.81); p=0.013 |
| **Ethnicity:** Asian | 0.61 (0.30-1.22); p=0.161 |
| **HIV acquisition mode:** MSM | (reference) |
| **HIV acquisition mode:** IDU | 2.08 (1.48-2.91); p<0.001 |
| **HIV acquisition mode:** Heterosexual | 1.64 (1.27-2.11); p<0.001 |
| **HIV acquisition mode:** Other | 1.32 (0.82-2.13); p=0.259 |
| **Smoking:** never | (reference) |
| **Smoking:** current smoking | 2.66 (2.10-3.37); p<0.001 |
| **Smoking:** past smoking | 1.38 (1.09-1.76); p=0.008 |
| **Education:** Mandatory School | (reference) |
| **Education:** Apprenticeship | 0.73 (0.58-0.91); p=0.006 |
| **Education:** Higher Education | 0.72 (0.55-0.94); p=0.017 |
| **Education:** Other/Missing | 0.77 (0.52-1.13); p=0.177 |
| **BMI:** Underweight | 3.38 (2.33-4.89); p<0.001 |
| **BMI:** Normal | (reference) |
| **BMI:** Overweight | 0.77 (0.63-0.94); p=0.010 |
| **BMI:** Obese | 0.74 (0.54-1.00); p=0.054 |
| **Hypertension** | 1.28 (1.07-1.53); p=0.008 |
| **Hepatitis C seropositivity** | 1.59 (1.20-2.10); p=0.001 |
| **Diabetes** | 1.87 (1.39-2.53); p<0.001 |
| **HIV RNA <50 copies/mL** | 0.52 (0.41-0.66); p<0.001 |

**Abbreviations.** BMI, body mass index; IDU, injection drug use; MSM, men who have sex with men

* leukocyte count 1 to 5 years before matching date
